# Systemic Uptake of Oxytetracycline and Streptomycin in Huanglongbing-Affected Citrus Groves after Foliar Application and Trunk Injection

**DOI:** 10.3390/antibiotics11081092

**Published:** 2022-08-12

**Authors:** Christopher I. Vincent, Faraj Hijaz, Myrtho Pierre, Nabil Killiny

**Affiliations:** 1 Horticultural Sciences Department, Citrus Research and Education Center, IFAS, University of Florida, Lake Alfred, FL 33850, USA; 2 Department of Plant Pathology, Citrus Research and Education Center, IFAS, University of Florida, Lake Alfred, FL 33850, USA

**Keywords:** Huanglongbing, citrus, oxytetracycline, streptomycin, adjuvant, foliar application

## Abstract

Huanglongbing (HLB), which is caused by the phloem-limited bacterium ‘*Candidatus* Liberibacter asiaticus,’ is an economically important disease of citrus in many regions of the world. Due to the significant damage caused by the HLB disease in recent years, the use of antibiotics was recommended for the therapy of this destructive disease. Products with active ingredients oxytetracycline and streptomycin have been approved for the control of the HLB via foliar application. However, previous work raised questions about the efficacy of foliar delivery of antibiotics in the field. In this study, we examined the effects of a variety of adjuvants on the uptake of oxytetracycline and streptomycin using the foliar application. We also compared the efficiency of foliar application of oxytetracycline and streptomycin with trunk injection. The ‘*Ca*. L. asiaticus’ titers in citrus plants were measured using quantitative PCR, and the levels of antibiotics were determined using the ELISA assay. Our results include extremely low levels of oxytetracycline and streptomycin in leaves that were covered during foliar application, indicating that neither streptomycin nor oxytetracycline was successfully systemically delivered by foliar application even after being mixed with adjuvants. Likewise, the ‘*Ca*. L. asiaticus’ titer0 was not affected by any of the foliar applications. High levels of streptomycin were detected in leaves that were exposed to direct foliar application, indicating that streptomycin was adsorbed or bound to citrus leaves. On the other hand, the trunk injection of oxytetracycline resulted in high levels of this antibiotic in leaves and significantly reduced the level of ‘*Ca.* L. asiaticus’ titer in citrus trees. Unfortunately, the trunk injection of streptomycin resulted in low levels of streptomycin in citrus leaves and did not affect the ‘*Ca.* L. asiaticus’ titer, indicating that streptomycin was either bound in the xylem of citrus trees or it was not applied in sufficient quantity required for the inhibition of *‘Ca.* L. asiaticus.’ Taken together, our current results demonstrated that foliar application of oxytetracycline and streptomycin did not effectively deliver antibiotics in citrus despite using adjuvants. Our results also suggested that oxytetracycline could be more effective against the HLB pathogen than streptomycin, which is possibly due to differences between the two in systemic movement in citrus trees.

## 1. Introduction

Huanglongbing (HLB; “citrus greening”) is currently considered the most destructive disease of citrus worldwide [1,2,3]. HLB is assumed to be caused by the uncultured, phloem-limited bacterium ‘*Candidatus* Liberibacter asiaticus’. HLB symptoms develop slowly, and with time, this disease inhibits the growth and yield by interfering with source–sink dynamics, which restrict the growth of roots, new shoots, and fruits [4,5,6]. Recently, HLB has become prevalent and endemic in Florida, Texas, and Brazil [7]. Because there are no resistant commercial cultivars to HLB [8], the use of antibiotics has been suggested to combat ‘*Ca.* L. asiaticus’ pathogen in plants [9].

Antibiotics have been effectively used for the control of many plant diseases for more than seventy years [10]. For instance, oxytetracycline has been used to control fire blight disease on pear and apple and bacterial spot disease on nectarine and peaches [11]. In addition, oxytetracycline has been used to control several plant pathogens including phytoplasmas, *Xanthomonas* spp., and *Pseudomonas* spp. [11]. Currently, only two antibiotics (streptomycin and oxytetracycline) are approved for use in citrus production. Three products with oxytetracycline or streptomycin as active ingredients were given emergency use approval in Florida in 2016, and they now have permanent labels for use via foliar application in citrus.

The initial use of antibiotics as a treatment for the HLB disease was proposed in the 1970s after it has been discovered that it was caused by a plant pathogen [11]. Early studies showed that tetracycline can suppress HLB symptoms when it is applied by trunk injection [12,13,14,15]. In addition, early works demonstrated that the foliar application of antibiotics was less efficient than trunk injection [16]. Recent studies have also cast doubts on the efficacy of foliar application in delivering antibiotics to the phloem, where the ‘*Ca.* L. asiaticus’ resides [17,18].

Previous studies showed that spiroplasmas were sensitive to several *antibiotics in vitro*. However, only tetracyclines were effective against these pathogens in plants, indicating that tetracyclines can be translocated to the phloem [19]. In a recent study, we investigated the uptake and distribution of streptomycin and oxytetracycline in citrus plants [20]. These two antibiotics were found in the xylem, phloem, leaves, and roots after stem delivery and root drench. The presence of these antibiotics in the phloem indicated that streptomycin and oxytetracycline could be effective against the HLB pathogen. The concentrations of these antibiotics in the canopy after stem treatment were higher than those detected after root drench [20]. On the other hand, the levels of antibiotics found in roots after root drench were higher than those found after stem delivery. The level of oxytetracycline detected in the leaves, xylem, and phloem was higher than that of streptomycin after root treatment [20]. On the other hand, the level of streptomycin in the roots was higher than that of oxytetracycline after root drench [20].

The efficacy of antibiotics in planta is highly affected by their uptake and translocation [19]. To understand the mechanism of oxytetracycline uptake and translocation, we studied the movement of oxytetracycline in girdled and non-girdled citrus seedlings and trees after root drench and trunk injection, respectively [21]. We found that oxytetracycline was present in the phloem and xylem below and above the girdle. This result indicated that the xylem was the main route for oxytetracycline movement [21]. The presence of oxytetracycline above the girdled area indicated that it was first translocated into the xylem and then was moved into the phloem [21].

Foliar spray is commonly used for the application of nutrients, insecticides, and herbicides. Unfortunately, most of the applied materials are deposited in the environment, and only a very small amount (<1%) reaches its target [22]. Soil drenching is also used for applying different agrochemicals including imidacloprid, which is used for the control of *D. citri.* Likewise, most of the applied materials are deposited in the soil, and only small amounts are taken up by the plants [23]. To minimize chemical loss during foliar spray and soil drenching, trunk injection has been developed as an alternative delivery method for agrochemicals. Trunk injection is considered superior to soil drenching because it delivers the exact dose, reduces deposition in the environment, and requires fewer applications [23]. However, trunk injection is not a common agricultural practice due to the cost of application. It is frequently used in urban areas where soil drench and foliar application are restricted [23,24].

To target phloem-limited pathogens, antibiotics applied using foliar application should be able to cross the leaf surface and travel through the plant vascular system. However, the presence of cuticles on the surface of plant leaves significantly reduces the rate at which applied chemicals can pass into the apoplast and subsequently into the vascular system. Uptake and loading into the phloem adds an additional subsequent limitation to systemic delivery in the phloem [25]. To enhance the uptake of agrochemicals by plant leaves, these chemicals are mixed with different types of adjuvants before being delivered using a foliar application [26].

In our recent study, we investigated the effect of nine commercial adjuvants on the uptake of oxytetracycline by citrus trees using foliar applications [24]. Our results showed that low levels of oxytetracycline (≈0.1 µg g^−1^) were detected in citrus leaves after being sprayed with aqueous oxytetracycline solution. Unfortunately, the mixing of adjuvants with the oxytetracycline solution did not improve its uptake by citrus leaves [24]. On the other hand, higher levels of oxytetracycline (≈6 µg g^−^^1^) were detected in leaves obtained from trunk-injected trees. In agreement with the chemical analysis, the ‘*Ca*. L. asiaticus’ titer was substantially diminished in trunk-injected trees one month after treatment, whereas it was not affected by any foliar application [24]. Interestingly, the uptake of oxytetracycline upon foliar application was enhanced by the perforation of citrus leaf cuticle, indicating that the citrus leaf cuticle was the main barrier against the uptake of oxytetracycline [24].

The physical properties of the compound such as the strength of acidity (pKa, how easily H^+^ ions are dissociated) and polarity or membrane permeability (log K_ow_) enable an initial forecast of its phloem translocation and distribution in plants [27,28]. Previous studies suggested that a compound with a pKa of 3.5–6.5 and Log K_ow_ of −0.5–3.5 is expected to be transported in the phloem, although these two properties interact in a non-linear fashion [25]. Based on these characteristics, oxytetracycline (pKa: 3.27, log K_ow_: −0.9) is expected to have a higher translocation rate than streptomycin (pKa: 10, log K_ow_: −7.5) in the phloem. In cases where delivery to meristematic or phloem tissues is the aim, phloem-translocated compounds have been found to be much more effective than those that are not [27,29]. In the current study, we assessed whether adjuvants could improve the delivery of streptomycin using the foliar application. In addition, we compared the efficiency of trunk injection and foliar application of streptomycin and oxytetracycline. Furthermore, we studied the degree to which these two antibiotics circulate within citrus plants as a proxy for systemic distribution to the phloem. We hypothesized that the trunk injection of streptomycin and oxytetracycline would be more effective against ‘*Ca*. L. asiaticus’ than foliar application and that oxytetracycline would be more effective than streptomycin.

## 2. Results

### 2.1. Effect of Adjuvants on the Uptake of Streptomycin (Study 1)

**Figure 1:**
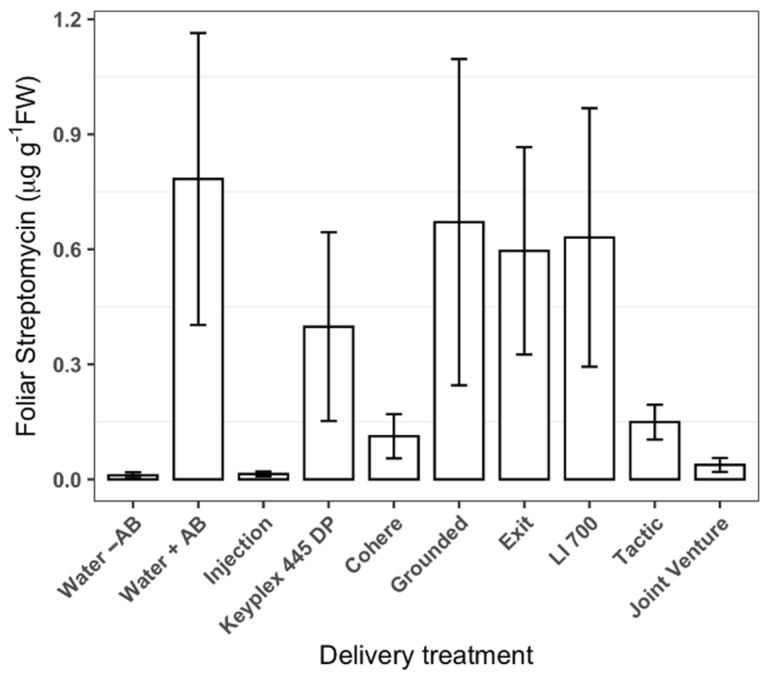
Concentrations of systemically delivered streptomycin in leaves of ‘Hamlin’ sweet orange (*C. × sinensis*) two days after delivery by the foliar application using various adjuvants or trunk injection. Values are concentrations in leaves that were covered to protect them from foliar sprays. The application dose was 0.78 g streptomycin per tree, which is equivalent to the labeled rate for foliar application. Bars represent means and error bars represent standard error (*n* = 12). The absence of labeling of treatments with different letters indicates that none are significantly different using Bonferroni’s protected least significant differences (*p* < 0.05). AB: anti-bacterial compound, in this case streptomycin. Bars not labeled with letters indicate that the treatment effect was not significant according to an analysis of variance.

High levels of streptomycin (8.1 ± 0.61 µg g^−1^ FWT) were detected in citrus leaves that were directly exposed to foliar applications (Table 1). On the other hand, low levels of streptomycin (0.33 ± 0.07 µg g^−1^ FWT) were detected in covered leaves upon foliar applications (Figure 1). The levels of streptomycin in covered leaves that were sprayed with streptomycin in the presence of adjuvants treatments were not significantly different from those that were treated with streptomycin solution or water (Figure 1). As Figure 1 shows, the variance of the streptomycin content in covered leaves was high relative to the means, indicating that the systemically delivered proportion was not statistically different from 0 µg g^−1^ FWT. Additionally, the concentrations of streptomycin in leaves obtained from trunk-injected trees were similar to those found in covered leaves of foliar-treated trees (Figure 1). No treatment achieved a mean greater than 1.92 µg g^−1^ FW in covered leaves, which is the minimum in plant effective concentration required for the inhibition of ‘*Ca.* L. asiaticus’ [30]. Uncovered leaves had nearly 20× the streptomycin concentration of covered leaves (Table 1). Neither the foliar application nor the trunk injection of streptomycin showed a significant decrease in ‘*Ca.* L. asiaticus’ (Table 2)

### 2.2. Comparison of Oxytetracycline and Streptomycin Delivery (Study 2)

**Figure 2:**
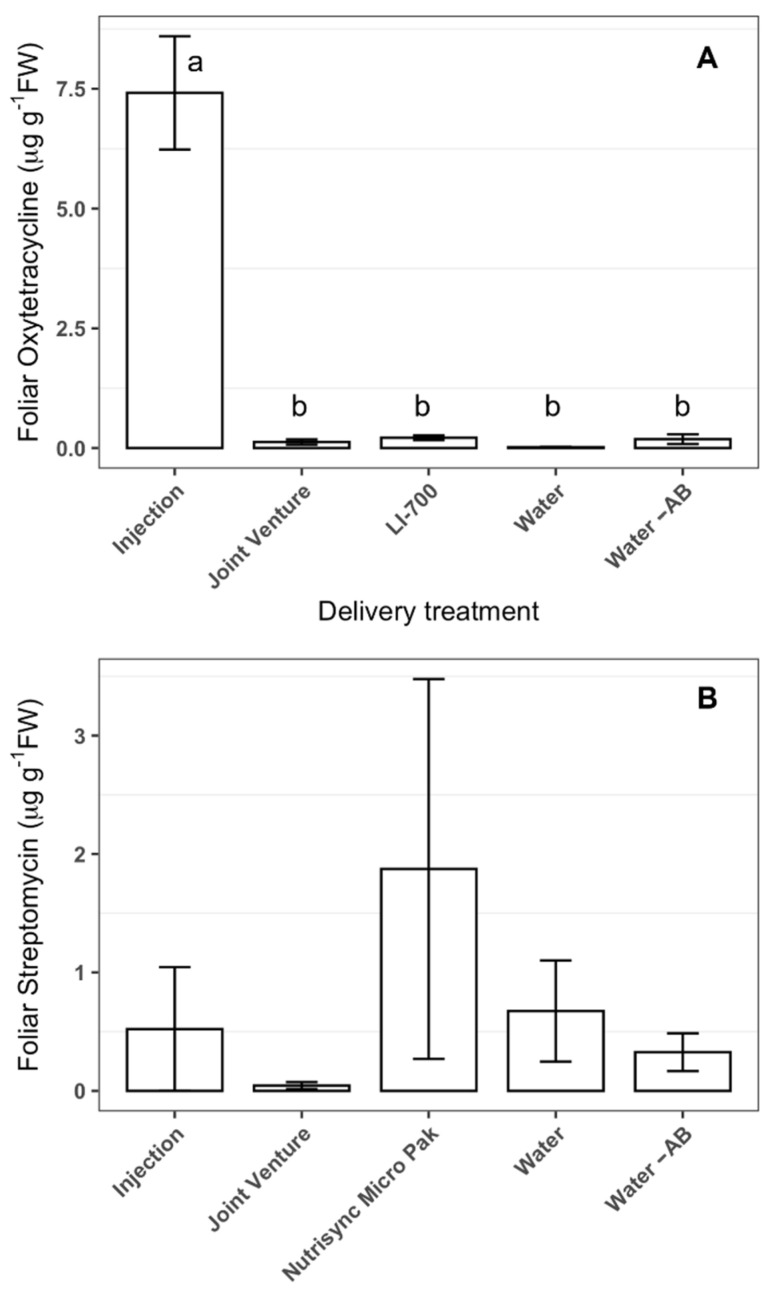
Concentrations of systemically delivered streptomycin (**A**) and oxytetracycline (**B**) in leaves of ‘Hamlin’ sweet orange (*C. × sinensis*) two days after delivery by the foliar application using various adjuvants or the trunk injection. Values are concentrations in leaves that were covered to protect them from direct contact with foliar sprays. The application dose was 0.78 g streptomycin per tree or 0.72 g of oxytetracycline per tree. Bars represent means and error bars represent standard error (*n* = 12). Treatments with different letters (a, b) are significantly different using Bonferroni’s protected least significant differences (*p* < 0.05). Bars not labeled with letters indicate that the treatment effect was not significant according to an analysis of variance. AB: anti-bacterial compound.

The average level (1.3 ± 0.42 µg g^−1^ FWT) of oxytetracycline in uncovered Hamlin leaves was similar to that of covered leaves (0.95 ± 0.34 µg g^−1^ FWT) upon foliar application (Table 1). Only the uncovered samples of Joint Venture and LI-700 resulted in greater foliar oxytetracycline than the water treatment (data not shown). The addition of adjuvants to the oxytetracycline solution did not result in a significant increase in its uptake in covered leaves upon foliar application (Figure 2). On the other hand, higher levels of oxytetracycline (≈7 µg g^−1^ FWT) were detected in leaves of Hamlin trees that were injected with oxytetracycline (Figure 2A). In the same manner, only the trunk injection of oxytetracycline showed a significant decrease in ‘*Ca.* L. asiaticus’ titer (*p* = 0.013).

The level of streptomycin in uncovered (10.7 ± 1.6 µg g^−1^ FWT) Hamlin leaves was significantly (*p* < 0.0001) higher than covered leaves (0.78 ± 0.27 µg g^−1^ FWT) upon foliar application (Table 1). None of the adjuvant treatments achieved higher streptomycin content in uncovered leaves than the streptomycin in water treatment (data not shown). In the same manner, none of the adjuvant treatments reached higher streptomycin content in covered leaves than those treated with aqueous streptomycin solution (Figure 2B). The level of streptomycin in covered and uncovered leaves upon treatment with aqueous streptomycin solution was not significantly different from those treated with water (Figure 2B). As in Study 1, trunk injection did not increase the streptomycin level over the adjuvant treatments (Figure 2B). In addition, no treatment achieved a mean greater than 1.92 µg streptomycin g^−1^ FW in covered leaves (Figure 2B), which is the minimum in plant effective concentration required for the inhibition of ‘*Ca.* L. asiaticus’ [30]. No significant decrease in ‘*Ca.* L. asiaticus’ titer was observed after trunk injection or the foliar application of streptomycin in this study.

## 3. Discussion

Our results showed that the addition of adjuvants to the foliar solution of streptomycin or oxytetracycline did not increase their systemic uptake by citrus leaves. In agreement with our current results, our previous results also demonstrated that mixing adjuvants with oxytetracycline solution did not improve its uptake by citrus leaves upon foliar application [24]. None of the foliar treatments used in this study resulted in concentrations that approached the minimum inhibitory concentrations of oxytetracycline or streptomycin as determined by [17,30]. Consistent with the chemical analysis results, the ‘*Ca*. L. asiaticus’ titer did not show any significant decline after any foliar treatment. Likewise, no significant decrease in the ‘*Ca*. L. asiaticus’ titer was observed in previous studies after foliar application of oxytetracycline, even in the presence of adjuvants [24]. These results indicated that foliar application, which is the only currently approved application method, is unlikely to reduce ‘*Ca.* L. asiaticus’ in citrus trees.

The uptake of sprayed agrochemical by plants depends on several factors including the selected adjuvant, plant species, and the type of chemical [31]. For example, the uptake of a copper fungicide was significantly enhanced through the isolated abaxial citrus leaf cuticle after the addition of the silicone-based L-77 surfactant [32]. However, no effect was observed on the uptake of copper through the isolated adaxial leaf cuticle, which lacks stomata. On the other hand, urea and petroleum oil adjuvants did not affect the uptake of copper by citrus leaves [32]. These results suggested that the abaxial leaf surface was more permeable than the adaxial leaf surface, which was possibly due to differences in the presence of stomata. In addition, the previous results also suggested that adjuvants may have minimal effects on the uptake of foliar-applied agrochemicals in the field because most of the applied material settles on the top surface of the leaves [24].

The citrus leaf cuticle provides a major barrier to the influx of foliarly applied compounds. In our previous work, we studied the cuticle structure of citrus leaves using transmission electron microscopy [24]. Our investigation showed that the top surface of the citrus leaf was covered with a thick (0.5–1.8 µm), uniform, and compact cuticle with no stomata. Early studies also showed that citrus leaf cuticle was thick and has very low permeability to water and hence resists the intake of liquids [33,34,35]. To check whether citrus cuticle was the main obstacle for the uptake of oxytetracycline by citrus leaves, we punctured the citrus leaf cuticle using laser light [24]. The levels of ‘*Ca.* L. asiaticus’ titer were significantly reduced in laser-perforated leaves after foliar application of oxytetracycline, whereas it was not affected in intact leaves [24]. The previous results suggested that only trace amounts of oxytetracycline were taken by intact citrus leaves due to the presence of the thick cuticle, which acts as a barrier. In the same manner, low levels of the fluorescent-labeled vancomycin and penicillin were taken up by intact citrus leaves, whereas laser perforation of the citrus cuticle significantly enhanced their uptake [36]. Laser perforation of the citrus cuticle also enhanced the uptake of other compounds including lysine, trehalose, and adenosine triphosphate (ATP) by citrus leaves [36]. The impact of perforation of the cuticle on the uptake of foliar-applied chemicals demonstrates the degree to which cuticular resistance limits the intake of chemicals to the mesophyll before they can reach the plant vascular system.

Our current results showed that trunk injection results in higher levels of oxytetracycline compared to the foliar application, which is in accordance with previous studies [24]. Our results also indicated that only trace amounts of oxytetracycline reach the vascular system of citrus plants after foliar application. Higher levels of oxytetracycline were detected in the xylem compared to the phloem after root drench and stem application [20]. This result indicated that the xylem was the primary route for the transportation of oxytetracycline. To test this hypothesis, we investigated the translocation of oxytetracycline in girdled citrus seedlings and trees after root drench and trunk injection, respectively [21]. The detection of oxytetracycline in the phloem and xylem tissues beyond the girdle confirmed that oxytetracycline was mainly translocated via the xylem [21]. In addition, the presence of oxytetracycline in the phloem tissues above the girdle indicated that it was first transported in the xylem and then moved to the phloem. In agreement with our previous findings, a bidirectional movement between the xylem and the phloem has been reported for several compounds [37]. Our previous results suggested that trunk injection of oxytetracycline was efficient for the delivery of oxytetracycline since it is mainly translocated in the xylem.

Although high levels of streptomycin were detected in uncovered citrus leaves that were directly treated with the foliar application, only small amounts were detected in covered leaves. Likewise, only trace amounts were detected in Hamlin citrus leaves after trunk injection of streptomycin. On the other hand, high levels of oxytetracycline were detected in citrus leaves after trunk injection. This result is similar to our previous results, which showed that the concentration of oxytetracycline in the roots of citrus seedlings was less than that of streptomycin after root drenching, indicating the greater translocation of oxytetracycline to the canopy [20]. On the other hand, the levels of oxytetracycline in the canopy were also higher than streptomycin after stem delivery and root drenching, indicating a greater translocation of oxytetracycline than of streptomycin [20]. High levels of streptomycin were also found in lower parts of tomato plants and peach seedlings upon root drench [38,39]. The previous results suggested that streptomycin may be adsorbed or bound to the xylem and other root tissues of citrus trees. The decrease in the concentration of streptomycin solution after the addition of crushed peach leaves also indicated that streptomycin binds to plant leaves [38]. The presence of high levels of streptomycin in uncovered leaves compared to covered leaves after foliar application also indicated that streptomycin was bound to citrus leaf tissues. In addition, the low level of streptomycin observed in citrus leaves after trunk injection also indicated that streptomycin was attached to the xylem of citrus trees. It is believed that the two guanido groups (positively charged) in streptomycin make it bind tightly to the xylem surface, which carries a negative charge [38]. The binding of streptomycin to plant tissues could decrease its translocation in plants. Although the mechanism is distinct, this result is also consistent with the model proposed by Kleier [28] for phloem-specific translocation, suggesting that increasing the polarity of xenobiotics decreased the likelihood that the compound would cross the cell membrane. Previous results showed that saturation of incubated tissues with streptomycin was required before it moves to other tissues [39]. The results indicated that higher concentrations of streptomycin may result in better translocation of streptomycin.

Our current study showed that streptomycin injection did not deliver significant concentrations to the canopy. In contrast to our results, a recent study showed that the injection of 2 g of streptomycin significantly reduced the ‘*Ca.* L. asiaticus’ titer in 3-year-old citrus trees [30]. However, the mean foliar concentration (1.71 µg g^−1^) at their peak was less than the minimum inhibitory concentration of 1.92 µg g^−1^ determined in the same study [30]. In the present study, we injected 0.78 g of streptomycin per tree (in a 6-year-old tree). Hence, the low levels of streptomycin obtained in this study after trunk injection can be attributed to the large size of citrus trees and low applied dose relative to Li et al. [30], although the applied dose in the present study is equivalent to the current recommended application rate per acre for foliar applications.

## 4. Material and Methods

### 4.1. Study 1: Effect of Adjuvants on Delivery of Streptomycin

#### 4.1.1. Plant Material

Five-year-old Hamlin sweet orange (*Citrus* × *sinensis* [L. ] Osbeck.) on Swingle citrumelo (×*Citroncirus* spp.) rootstock trees was used on June 6, 2020 for the adjuvant delivery of streptomycin in the study. The location was in Lake Alfred, FL, USA (28.09° N, 81.37° W, elevation 51 m a.s.l.). Trees were growing in sandy soil, and each tree was irrigated daily with a half-gallon using a drip irrigation system.

#### 4.1.2. Experimental Design

The experiment was executed in a randomized complete block design with 12 blocks and 10 treatments. Blocks were arranged linearly within rows, with two blocks per row. Six rows were used for the experiment. The distance between planted trees within the row was 2.75 m and that between rows was 6 m (approximately 618 trees per ha). The experimental unit was one tree, and we left two buffer trees between each treated tree to avoid cross-contamination.

#### 4.1.3. Treatments

This study included 10 treatments: adjuvants (1) Exit, (2) Keyplex, (3) Grounded, (4) Cohere, (5) Tactic, (6) Joint Venture, (7) LI 700 or (8) streptomycin trunk injection, (9) foliar application of streptomycin in water (no adjuvant), and (10) a negative control that was sprayed with water (no streptomycin). For foliar application, 1.56 g of FireWall 50WP (0.78 g streptomycin; AgroSource, Tequesta, FL, USA) was dissolved in 1.25 L water and applied to each tree (approximately applied to runoff), using a CO_2_-pressurized hand sprayer at approximately 100 psi. For the injection treatment, 1.56 g of FireWall 50WP was dissolved in 20 mL of water and injected above the graft union as described in our earlier study [24]. The applied rate (0.78 g streptomycin per tree) was equivalent to the label-recommended rate of 11 oz acre^−1^ (771 g ha^−1^). Adjuvant rates were used at the maximum labeled rates as described in our previous study [24].

Before foliar application, one shoot of each tree was flagged and labeled for leaf sampling, and one young leaf of each labeled branch was collected for ‘*Ca*. L. asiaticus’ pre-sampling analysis. Before treatment application, one shoot in each tree was covered in a plastic bag, and the bag was removed after the applied treatment had dried. Two days after application, 3 leaves were collected from uncovered shoots and another 3 leaves were collected from shoots that were covered with plastic bags. One month after application, one young leaf of the same labeled branches was collected to measure the detection of ‘*Ca*. L. asiaticus.’

### 4.2. Study 2: Comparison of Streptomycin and Oxytetracycline Delivery

#### 4.2.1. Plant Material

Trees from the same planting, although not the same trees, were selected for treatment on 13 October 2020, to study the adjuvant delivery of streptomycin and oxytetracycline. Grove care was the same as in Study 1.

#### 4.2.2. Experimental Design

The experiment was also executed in a randomized complete block design with 12 blocks and 9 treatments (4 with oxytetracycline, 4 with streptomycin, and one negative control). The arrangement of blocks in rows and experimental units were the same as in Study 1. This study included 9 treatments: (1) Joint Venture + oxytetracycline, (2) LI 700 + oxytetracycline, (3) water + oxytetracycline, (4) Nutrisync Micro Pak + streptomycin, (5) Joint Venture + streptomycin, (6) LI 700 + streptomycin, and (7) water + streptomycin, (8) trunk injection of oxytetracycline, (9) trunk injection of streptomycin. The concentrations of streptomycin used in this study were the same as those used in Study 1. Four grams of FireLine 17 WP (AgroSource; 0.72 g oxytetracycline) were applied in 1.25 L per tree (approximately to runoff), including the no-adjuvant control with water as described in Study 1. For the injection treatment, 4 g of FireLine 17 WP were dissolved in 20 mL of water and injected using the same approach as in Study 1. The applied rate (0.72 g oxytetracycline per tree) was equivalent to the labeled rate of 24 oz acre-1 (1681 g ha^−1^). Adjuvants were used at the maximum labeled rates as in [24]. Labeling, sampling, and application were the same as implemented in Study 1. Leaf sampling for antibiotic quantifications and ‘*Ca.* L. asiaticus’ titer estimation was performed as described above in Study 1. In addition, before treatment application, one shoot in each tree was covered using a plastic bag, which was removed 24 h after application.

#### 4.2.3. Extraction and Analysis of Oxytetracycline and Streptomycin

The extraction and analysis of streptomycin and oxytetracycline were performed as described in our previous studies [20,24]. Streptomycin and oxytetracycline ACCEL ELISA kits were obtained from Plexense, Inc. (Davis, CA, USA) and were used according to the manufacturer’s instructions [20,24]. The quantitation ranges of the streptomycin and oxytetracycline kits were 0.150–12.5 ng mL^−1^ and 1.56–50 ng mL^−1^, respectively.

#### 4.2.4. ‘*Ca*. L. asiaticus’ Detection

DNA was extracted using potassium acetate buffer as described in our previous study [24]. Extracted DNA was adjusted to 100 ng/μL and then used for RT-qPCR amplification using a TaqMan Universal PCR master mix (Life Technologies, Carlsbad, CA, USA) and degenerate genus-specific (rpoB) primer-probe sets [24]. Assays were performed using an Applied Biosystems QuantStudio 3 Real-Time PCR system (Applied Biosystems, Foster City, CA, USA). (Ct). The qPCR cycle threshold (Ct) values ≤ 35 were assigned as positive for ‘*Ca*. L. asiaticus’ infection, whereas qPCR Ct values > 35 were assigned as negative [24].

#### 4.2.5. Statistical Analysis

Data were analyzed using analysis of variance of mixed linear models, in which block (replication) was included as a random effect using the lm command in base R. Treatment was included as a fixed effect, and the sample (covered or uncovered leaf) was included as a fixed effect nested within the plant. For significant effects, least significant differences were determined via Bonferroni’s protected LSD, using the lsd.test command in the {agricolae} R package [40,41].

## 5. Conclusions

**Figure 3:**
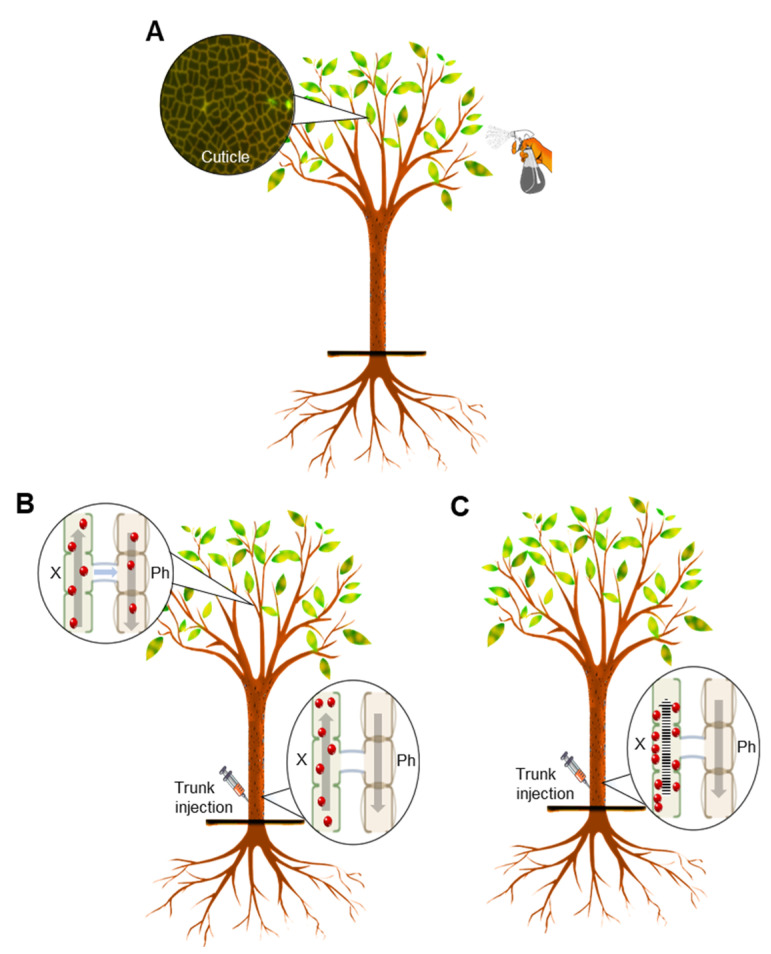
Illustration of efficacy of the different delivery methods for oxytetracycline and streptomycin to citrus trees. (**A**) Foliar application failed to deliver oxytetracycline and streptomycin due to the thickness of the leaf cuticle. (**B**) Oxytetracycline was successfully delivered to foliage after trunk injection. It moves upward via the xylem to the canopy (unidirectional), where it is translocated into the phloem (bidirectional) and distributed to leaves. (**C**) Streptomycin adheres to the cells after trunk injection and is not translocated. Red dots represent anti-microbial compounds. X: xylem. Ph: phloem.

In summary, our results showed that, consistent with our hypotheses, neither oxytetracycline nor streptomycin was successfully delivered by the foliar application even after mixing these antibiotics with adjuvants (Figure 3A). This conclusion was based on the trace levels of oxytetracycline and streptomycin that were detected in covered leaves, which indicated the low systemic delivery of these antibiotics after foliar application (Figure 3A). This conclusion was also supported by the PCR results, which did not show any decrease in ‘*Ca.* L. asiaticus’ titer after any foliar application. The high levels of streptomycin in citrus leaves (uncovered) that were directly exposed to the foliar application indicated that streptomycin was adsorbed or bound within the leaves. The failure of the foliar application to deliver oxytetracycline and streptomycin could result from the thick cuticle, which covers the citrus leaf and acts as a barrier to xenobiotics. On the other hand, our results showed that trunk injection was an effective delivery method for oxytetracycline (Figure 3B). This conclusion is supported by the high levels of oxytetracycline and the low levels of ‘*Ca.* L. asiaticus’ titer found in citrus leaves after trunk injection (Figure 3B). Unfortunately, a low level of streptomycin was detected in citrus leaves after trunk injection (Figure 3C). In agreement with the ELISA results, no significant decrease in ‘*Ca.* L. asiaticus’ titer’ was observed after trunk injection of streptomycin. This result indicates that injected streptomycin is bound to the xylem of citrus trees and is not easily moved systemically, even after trunk injection.

## Figures and Tables

**Table 1:** Concentrations of streptomycin and oxytetracycline in covered or directly sprayed leaves of ‘Hamlin’ sweet orange (*C. × sinensis*) after foliar application in two different adjuvant studies.

Study	Compound	Rate per Tree	Antimicrobial Concentration (µg g^−1^ FW)	
			Covered Leaves	Sprayed Leaves
Streptomycin—9 adjuvants	Streptomycin	0.78 g	0.33 ± 0.07 ^b^	8.1 ± 0.61 ^a^
Combined—4 adjuvants	Streptomycin	0.78 g	0.78 ± 0.27 ^b^	10.7 ± 1.6 ^a^
Combined—4 adjuvants	Oxytetracycline	0.72 g	0.95 ± 0.34 ^a^	1.3 ± 0.42 ^a^

Means with different letters are significantly different using a two-tailed student *t*-test (*p* < 0.05).

**Table 2:** Cycle threshold values for detection of ‘*Candidatus* Liberibacter asiaticus’ DNA in leaves of ‘Hamlin’ sweet orange (*C. × sinensis*) before and after foliar application or trunk injection of two different adjuvant studies. *P*(T) represents a two-tailed paired *t*-test. Post-treatment sampling was 1 month after application.

Study	Anti-Microbial Compound	Treatment	Pre-Treatment		Post-Treatment		*P*(T)
			Mean	±SE	Mean	±SE	
Streptomycin only	Streptomycin	Cohere	31.3	0.72	31.9	0.67	0.41
		Exit	31.2	0.92	31.0	0.62	0.86
		Grounded	29.7	0.67	30.1	0.54	0.59
		Joint Venture	29.9	0.66	31.8	0.67	0.14
		Keyplex 445 DP	32.3	0.76	31.2	0.51	0.27
		LI 700	30.7	0.95	31.0	0.59	0.82
		Tactic	30.5	0.72	30.9	0.48	0.73
		Injection	30.5	0.86	31.2	0.77	0.55
		Water − AB	30.3	0.83	30.3	0.77	0.92
		Water + AB	30.5	0.74	32.0	0.46	0.12
	None	Water − AB	30.3	0.83	30.3	0.77	0.92
Oxytetracycline and Streptomycin	Streptomycin	Injection	29.8	1.25	30.8	0.83	0.87
		Joint Venture	30.4	0.60	30.8	0.68	0.75
		Nutrisync Micro Pak	31.3	0.59	30.4	0.68	0.37
		Water	29.7	0.40	30.3	0.72	0.41
	Oxytetracycline	Injection	30.7	0.61	29.8	0.98	0.002
		Joint Venture	30.4	0.92	31.0	0.52	0.47
		LI 700	29.8	0.64	30.8	0.68	0.19
		Water	29.3	0.86	30.7	0.66	0.17
	None	Water	29.8	0.88	29.7	0.71	0.82

## Data Availability

Data may be made available upon reasonable request to the corresponding author.
